# Study on Crack Development and Micro-Pore Mechanism of Expansive Soil Improved by Coal Gangue under Drying–Wetting Cycles

**DOI:** 10.3390/ma14216546

**Published:** 2021-11-01

**Authors:** Hongxing Zhu, Yan Zhang, Zhuhan Li, Xiaoyu Xue

**Affiliations:** 1 College of Energy and Transportation Engineering, Inner Mongolia Agricultural University, Hohhot 010018, China; zhuhongxing@emails.imau.edu.cn (H.Z.); xuexiaoyu@emails.imau.edu.cn (X.X.); 2 School of Planning, University of Waterloo, Waterloo, ON N2L 3G1, Canada; z2246li@uwaterloo.ca

**Keywords:** expansive soil, cracks, drying–wetting cycles, coal gangue powder, micro-pore mechanism, roughness

## Abstract

Expansive soil is prone to cracks under a drying–wetting cycle environment, which brings many disasters to road engineering. The main purpose of this study is use coal gangue powder to improve expansive soil, in order to reduce its cracks and further explore its micro-pore mechanism. The drying–wetting cycles test is carried out on the soil sample, and the crack parameters of the soil sample are obtained by Matlab and Image J software. The roughness and micro-pore characteristics of the soil samples are revealed by means of the Laser confocal 3D microscope and Mercury intrusion meter. The results show that coal gangue powder reduces the crack area ratio of expansive soil by 48.9%, and the crack initiation time is delayed by at least 60 min. Coal gangue powder can increase the internal roughness of expansive soil. The greater the roughness of the soil, the less cracks in the soil. After six drying–wetting cycles, the porosity and average pore diameter of the improved and expanded soil are reduced by 37% and 30%, respectively, as compared to the plain expansive soil. By analyzing the cumulative pore volume and cumulative pore density parameters of soil samples, it is found that the macro-cracks are caused by the continuous connection and fusion of micro-voids in soil. Coal gangue powder can significantly reduce the proportion of micro-voids, cumulative pore volume, and cumulative pore density in expansive soil, so as to reduce the macro-cracks.

## 1. Introduction

Expansive soil is a type of high plastic clay rich in high hydrophilic montmorillonite, illite, and other clay minerals. Compared with other ordinary clays, it has water swelling and water loss shrinkage characteristics. These characteristics make the areas with expansive soil prone to cracks and instabilities under the repeated alternating effects of rainwater infiltration and atmospheric evaporation, which brings many serious security risks to roads, water conservancy, farmland, and other engineering facilities [1,2,3,4,5]. In recent years, many scholars have begun to study the development of cracks in expansive soil, by using relevant computer technologies and image recognition technologies to extract cracks, and then quantitatively analyzing the parameters of the extracted cracks, in order to obtain the related characteristics of soil crack development [6,7,8]. In order to further explore the causes of soil cracking, some scholars have studied the surface roughness of cracks and found that there is an inverse relationship between the roughness and the crack width. With the increase in the roughness coefficient, the normal stress on the crack surface increases gradually, while the peak value of the initial shear stress increases as well [9,10]. Under the influence of the alternations of drying and wetting, the pore change, geometric shape, spatial distribution, and connectivity in the soil have great influences on the soil structure and crack evolution [11,12]. In recent years, more and more studies have been carried out on the micro-characteristic parameters of soils, such as porosity, average pore diameter, pore volume, and other parameters. The main methods to detect pore characteristic parameters are the mercury intrusion method, the scanning electron microscope method, and the nitrogen adsorption method [13,14]. Some scholars have further classified the pores by the pore size, to further analyze the pores quantitatively [15]. In order to reduce the harms of expansive soil in engineering construction, some scholars use materials (such as cement, lime, biological enzyme, and industrial waste residue, etc.) to modify expansive soil [16,17,18,19,20,21]. Li et al. [22] improved the expansive soil with sand, and studied the effects of sand content and particle size on the development of expansive cracks. The results show that the crack rate of soil sample decreases gradually with the increase in sediment content and particle size. Zhang et al. [23] improved the expansive soil by compounding cement and lignin. It was found that the hydration reaction and gel reaction of cement in expansive soil resulted in the formation of high strength materials, which increased the strength of expansive soil. An appropriate amount of lignin can be filled in the soil pores to make the soil particles more closely connected, so as to improve the stability of expansive soil. Chu et al. [24] studied the effects of drying–wetting cycles on the expansion behavior and compressibility of iron tailing sand and calcium carbide slag-modified expansive soil. The micro-pore structure of soil samples was analyzed by the mercury injection method. Kumar et al. [25] treated expansive soil with lime to improve its stability. Farzaneh et al. [26] studied the effect of hollow polyester fiber as an additive on the free expansion rate of expansive soil. The results show that the higher the fiber density, the smaller the free expansion rate of the soil sample. However, there is still a lack of consideration on the cost of improved materials, the degree of difficulty in obtaining the sample, the yield, and other factors. As a kind of waste associated with the process of coal production, there is a huge annual production of coal gangue in China. Long-time stacking of coal gangue will pollute the environment and occupy a large amount of land, resulting in a waste of resources [27,28]. Relevant studies show that coal gangue can be used as road-building materials and soil improvement materials [29]. Some scholars have studied the application of coal gangue in road construction and found that coal gangue has a high bearing capacity, which can meet the requirements of road construction materials [30]. Coal gangue also contains many high valence cations, which can react with low valence cations in expansive soil to form more stable compounds [31]. Therefore, using coal gangue to improve expansive soil can not only improve the stability of expansive soil subgrade, but also reduce the environmental pollution caused by coal gangue [32,33]. To date, most scholars have only studied the development of soil cracks, but there is still a lack of research on the micro-pore evolution mechanism of macro-crack development and the influence of crack surface roughness on crack development.

In this work, different amounts of coal gangue powder are used to modify expansive soil, and the optimal amount of coal gangue powder is determined by the expansion rate tests. The drying–wetting cycle tests of the improved expansive soil and plain expansive soil were carried out to study the development law of cracks. The roughness parameters of soil samples are measured by the Laser confocal 3D microscope, and the micro-pore change parameters of soil samples under drying–wetting cycles are measured by the mercury injection test. The effects of soil roughness and micro-pore characteristics on crack development are analyzed, respectively.

## 2. Materials and Methods

**Figure 1:**
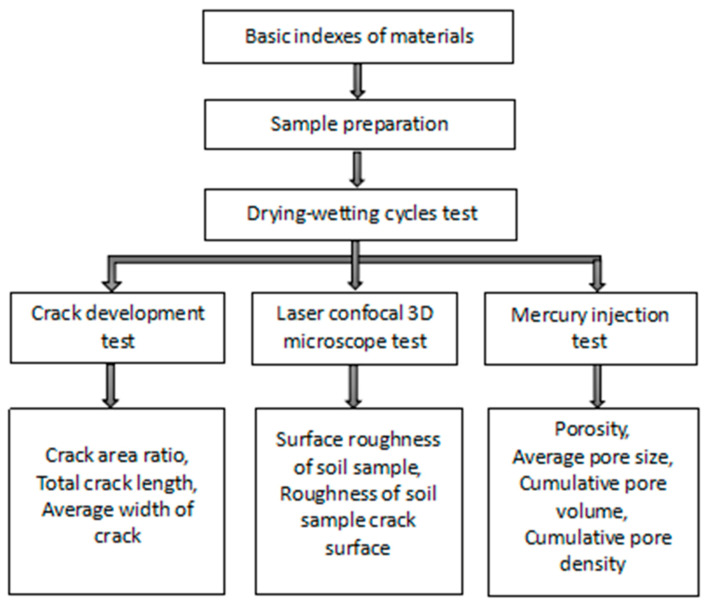
Flowchart of test design method.

In this section, the basic indexes and test methods of the materials used will be introduced. The test flowchart is shown in Figure 1.

### 2.1. Expansive Soil

The expansive soil used in this study was taken from Gaomiaozi Township, Xinghe County, Inner Mongolia, which is a gray and white powder with fine texture. The basic physical indexes of expansive soil are in accordance with the test procedure [34] and ISO/DIS 20290-1 [35], as shown in Table 1.

The basic chemical composition of expansive soil is detected by XRF (EXF-90 Shenzhen hongyongjingyi Technology Co., Ltd., Shenzhen, China), as shown in Table 2.

**Figure 2:**
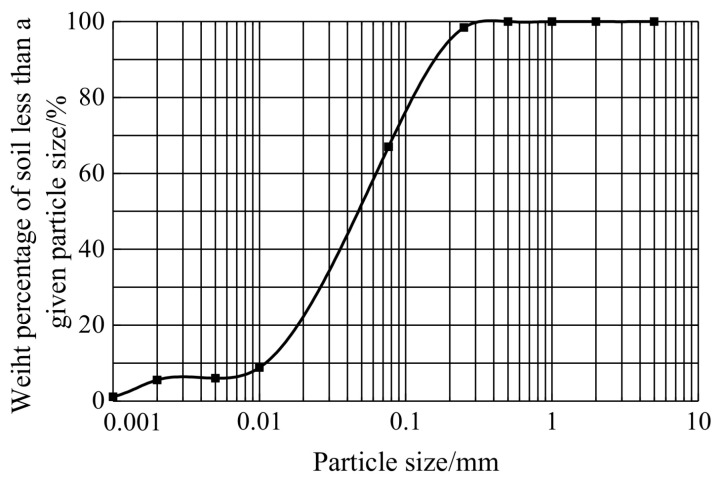
Particle analysis curve of expansive soil.

The particle-grading curve of expansive soil is obtained according to the standard method in reference [34], as shown in Figure 2.

According to the data in Figure 1, two gradation indexes of soil can be calculated. The non-uniformity coefficient is C_u_ = 6 > 5, and the curvature coefficient is C_C_ = 0.7 ≠ 1~3. It can be judged that the soil particle size distribution is poor [34].

### 2.2. Coal Gangue

The coal gangue used in this test was collected from the Gongwusu No.4 mining area of Shenwu coalmine in Wuhai City. It is black, gray, massive, and hard in texture. It belongs to carbonaceous shale. The basic physical indexes of coal gangue were measured according to the test procedure [34] and ISO/DIS 20290-1 [35], as shown in Table 3.

The basic chemical composition of expansive soil is detected by XRF (EXF-90 Shenzhen hongyongjingyi Technology Co., Ltd., Shenzhen, China), as shown in Table 4.

### 2.3. Sample Preparation

A total of 0%, 3%, 5%, 7%, 9%, 11%, and 13% coal gangue powder were mixed into expansive soil, and then compaction tests were carried out on these seven kinds of soil samples. Through the compaction tests, the optimal moisture contents were 22.3%, 22.4%, 22.5%, 23.4%, 23.2%, 23.1%, and 22.7%, respectively. The results show that when the content of coal gangue powder is 9%, the no-load expansion rate of expansive soil reaches a minimum of 9.6%. For these seven kinds of soil samples, the free expansion rate tests are carried out with measuring cylinders, and the cylinder scale is recorded every 5 h until the volume no longer changes. As a result, the free expansion rates of the seven kinds of soil samples are 47.3%, 44.6%, 42.4%, 39.1%, 34.3%, 34.5%, and 35.2%, respectively. When the coal gangue powder content is 9%, the free expansion rate of the sample is 34.3%, and the sample has changed from expansive soil to non-expansive soil [36]. In this study, 9% coal gangue powder was used as the optimal water content to carry out the crack development test and the mercury injection test.

### 2.4. Testing Methods

#### 2.4.1. Drying–Wetting Cycles Test

The plain expansive soil and 9% coal gangue powder-improved expansive soil were sprayed with water and evenly stirred until the water content reached their respective optimal water contents, and each mixture was stewed for 24 h to render the water evenly distributed. The compacted mixture was compacted in a ring cutter with a diameter of 61.8 mm and a height of 20 mm. The degree of compaction was 93%, which was divided into two groups. One group was used for the crack development test under drying–wetting cycles, and another group was used for the mercury injection test. The self-made dripping device is used in the drying–wetting cycle device to simulate the rainy weather. A permeable stone is placed above and below the ring cutter specimen to simulate the subgrade seepage effect. After the specimen is completely saturated, the specimen is taken out for natural air-drying for 24 h. The surface of the soil sample is photographed at intervals to observe the crack development. At this point, the first drying–wetting test is completed, and it is repeated until the sixth cycle.

#### 2.4.2. Crack Development Test

**Figure 3:**
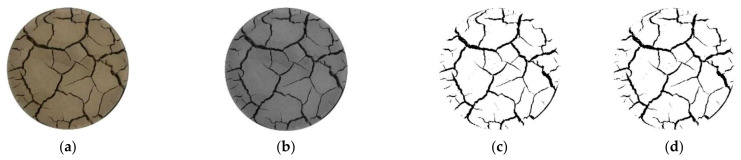
Crack image processing process: (**a**) original image; (**b**) gray processing; (**c**) binary processing; (**d**) noise reduction treatment.

Photos are taken of soil samples under the drying–wetting cycle, and attention is paid to the consistency of height and angle. In order to calculate the crack parameters more accurately, the image needed to be further processed. In this study, the original colour image was grayed by the Matlab software, and the grayed image is binarized. The black part represents the crack, and the white part represents the cracked soil. As some non-crack black spots will appear on the surface of the image after binarization, it is necessary to reduce the noise by using the Adobe Photoshop software. Next, the area ratio of cracks is calculated by the Matlab software program, and the total length and average width of cracks after binarization are measured by using the Image J software. The measurement method first calibrates the size when taking photos, then inputs the calibrated size and unit into the Image J program, and finally measures the soil sample cracks. The crack image processing process is shown in Figure 3.

#### 2.4.3. Micro-Characteristic Test

**Figure 4:**
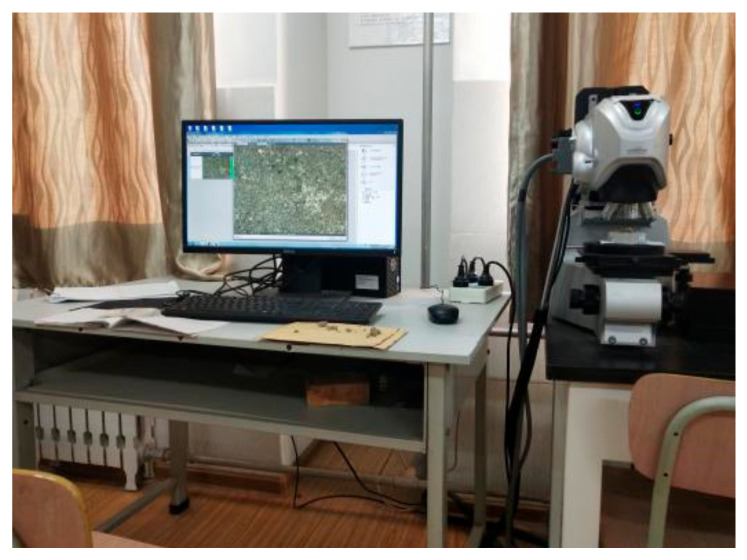
VK-X150 Laser 3D microscope.

**Figure 5:**
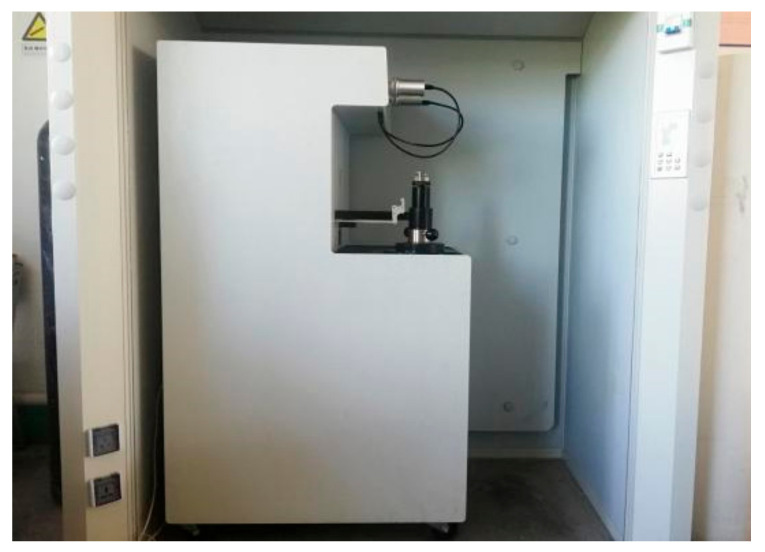
Auto pore 9500 mercury intrusion meter.

After six drying–wetting cycles, the crack surface of the crack-developed sample is cut open, and the crack surface and the surface of the soil sample are scanned with the Laser confocal 3D microscope (VK-X150, Keyence Co., Ltd., Osaka, Japan), as shown in Figure 4, to obtain roughness parameters to analyze the relationship between roughness and crack development. By using a sampler, a cube of 1 cm × 1 cm × 1 cm soil was cut in the center of another set of specimens as a sample for the mercury injection test under different drying and wetting cycles in a fully automatic mercury injection apparatus (Autopore IV 9510, Micromeritics Co., Ltd., Norcross, GA, USA), as shown in Figure 5. The mercury injection test was carried out to determine soil porosity, average pore diameter, cumulative pore volume, and cumulative pore density parameters.

During the mercury intrusion test, when the normal force component and the tension direction on the contact surface are the same, the amount of mercury is pressed into the equilibrium state, which indicates that the mercury injection process has stopped. The capillary model washborn [37] is used for pore size analysis, and the washborn equation is derived from the Young–Laplace formula, as shown in Equation (1) below.

(1) $$P=-\frac{4\gamma \cos\theta }{R}$$

where P is the mercury inlet pressure (kPa). γ is the surface tension of mercury (N/m), and the value is 0.48 N/m. θ is the contact angle of mercury to the material and soil particles, and the value is 140°. R is the pore diameter (μm).

## 3. Results and Analysis

### 3.1. Crack Development Process of Expansive Soil

**Figure 6:**
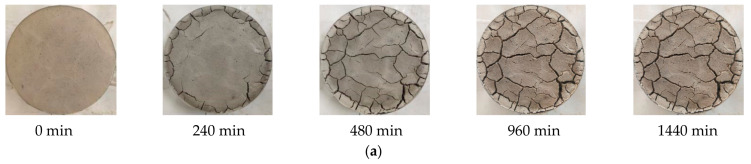

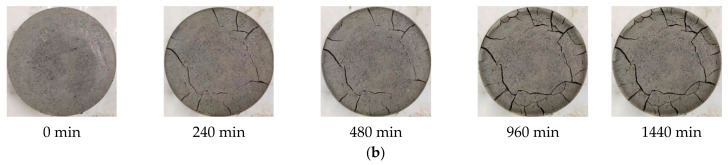
Crack development process of a test piece under natural air-drying conditions: (**a**) crack development process of plain expansive soil; (**b**) crack development process of improved expansive soil.

As there are many pictures, the crack development process of plain expansive soil and the improved expansive soil after the sixth drying–wetting cycle is only shown here, as shown in Figure 6.

#### 3.1.1. Crack Area Ratio

**Figure 7:**
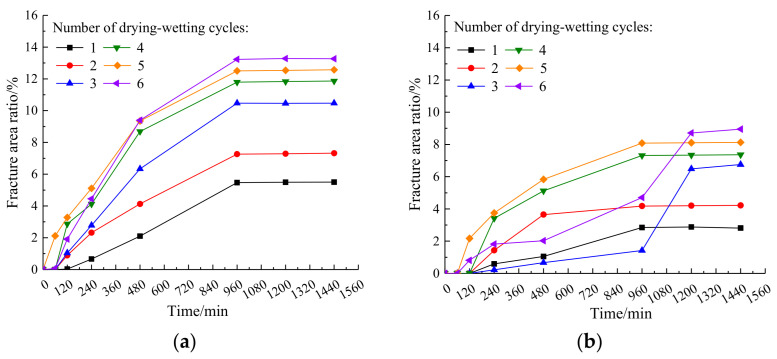
Variation of crack area ratio of plain expansive soil and improved expansive soil with time under a drying–wetting cycle: (**a**) plain expansive soil; (**b**) improved expansive soil.

The variation of the crack area ratio of plain expansive soil and the improved expansive soil with time under the drying–wetting cycle is shown in Figure 7.

It can be observed in Figure 7a that the crack area ratio of plain expansive soil increases as the amount of the drying–wetting cycle increases. In the first four drying–wetting cycles, the cracks begin to appear at 120 min. In the fifth and sixth drying and wetting cycle, the cracks begin to appear at 60 min. These results demonstrate that the crack area ratio increases rapidly between 120 and 960 min, and the increasing speed of the crack area ratio increases with the increase in the drying–wetting cycles. In the period of 960–1440 min, the area ratio of cracks remains unchanged, which indicates that there are some strong instabilities in the early stages of plain expansive soil; therefore, the soil can be easily damaged, due to the influence of the drying–wetting cycles. It can be observed from Figure 7b that the crack area ratio and development speed of the improved expansive soil under each drying–wetting cycle is significantly lower than that of the plain expansive soil. For the first four drying–wetting cycles, the cracks begin to appear at 240 min, while during the fifth and sixth drying–wetting cycles, the cracks begin to appear at 120 min. This indicates that the coal gangue powder can effectively delay the initial crack germination time of the expansive soil. After a comparative analysis, the crack area ratio of the improved expansive soil decreased 48.9%, with the largest decrease found during the first drying–wetting cycle. Compared with the plain expansive soil, the crack germination time is delayed by at least 60 min.

#### 3.1.2. Total Crack Length

**Figure 8:**
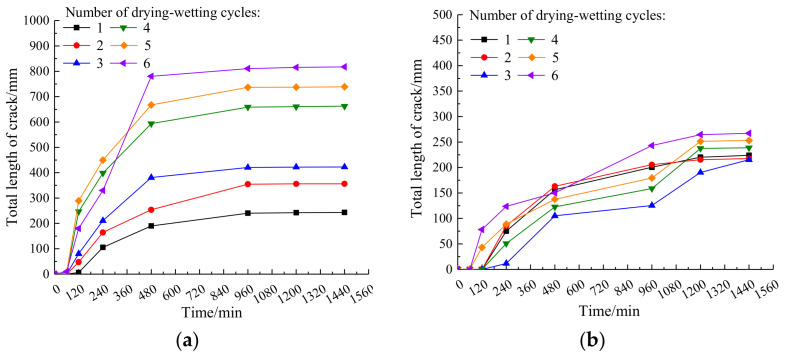
Variation of total crack length of plain expansive soil and improved expansive soil with time under drying–wetting cycle: (**a**) plain expansive soil; (**b**) improved expansive soil.

The variation of total crack length of plain expansive soil and the improved expansive soil with time under different drying–wetting cycles is shown in Figure 8.

It can be observed in Figure 8a that the total crack length curve of plain expansive soil changes with the increase in drying–wetting cycles. When the time is between 60 and 480 min, the total crack length increases rapidly. When it is between 480 and 960 min, the total crack length increases slowly. When it is between 960 and 1440 min, the total crack length tends to be stable. Finally, after six cycles of drying–wetting, the total length of the crack increases by 70.2%, as compared with the first one. It can be observed from Figure 8b that the total crack length of the improved expansive soil is significantly smaller than that of the plain expansive soil, and the distribution of each curve is closer than the increase in the number of drying–wetting cycles, which further indicates that the stability of the improved expansive soil is significantly improved. In each drying–wetting cycle, the growth rate of the total crack length obviously decreases, and coal gangue powder can effectively restrain the increase in the crack length of expansive soil.

#### 3.1.3. Average Width of Crack

**Figure 9:**
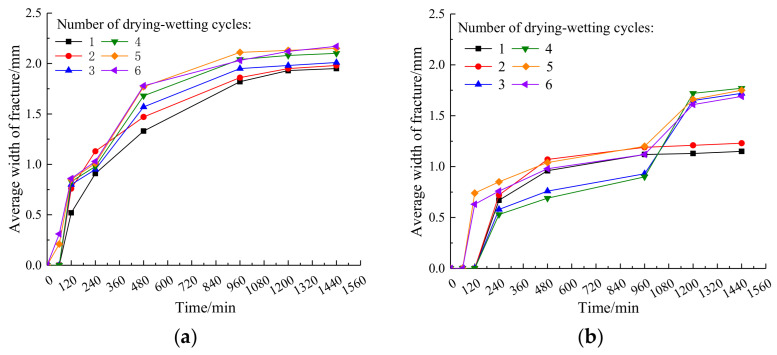
Variation of average crack width with time in plain expansive soil and improved expansive soil: (**a**) plain expansive soil; (**b**) improved expansive soil.

The relationship between average crack width and time of plain expansive soil and plain expansive soil under different drying–wetting cycles is shown in Figure 9.

It can be observed from Figure 9a that the influence of drying–wetting cycles on the average width of cracks in plain expansive soil is small, and the distribution of each curve is relatively dense. After six drying–wetting cycles, the average width of cracks in plain expansive soil increases by 10.1% compared with that in the first drying–wetting cycle. It can be observed from Figure 9b that the average crack width of the improved expansive soil increases rapidly at first and then tends to be stable in the first two drying–wetting cycles, and its variation range is small. In the third and fourth drying–wetting cycles, the average crack width in the early stage is smaller than that in the first and second drying–wetting cycles. It begins to increase rapidly between 960 and 1200 min and finally tends to be stable between 1200 and 1440 min. In the fifth and sixth drying–wetting cycles, the average crack width is larger in the early stage, and it also increases rapidly between 960 and 1200 min, and finally tends to be stable between 1200 and 1440 min. This is because of the brittleness of the improved expansive soil at the early stage of the drying–wetting cycle, which leads to the rapid increase in crack width when the soil sample is drying. The skeleton of the soil sample is destroyed to a certain extent, and the coal gangue powder particles are separated from the expansive soil particles, which lead to the rapid increase in crack width again in the later stage. Overall, the average crack width of expansive soil, improved by coal gangue powder, is smaller than that of plain expansive soil.

### 3.2. Roughness of Crack Surface Analysis

**Figure 10:**
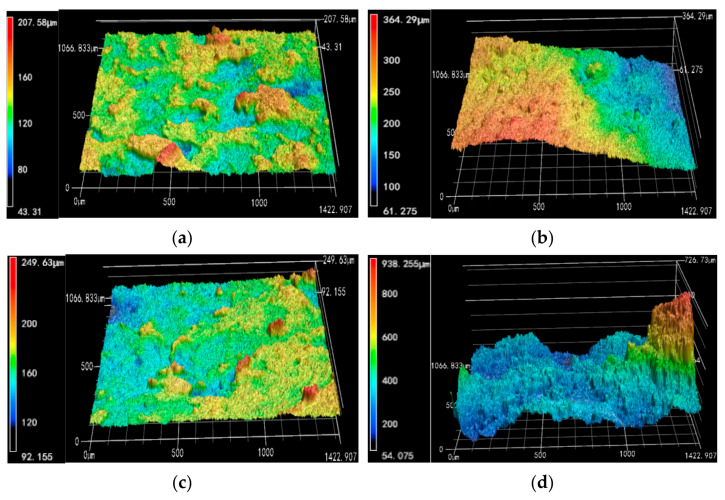
3D images of the surface and crack surfaces of plain expansive soil and improved expansive soil: (**a**) 3D elevation map of plain expansive soil sample surface; (**b**) 3D elevation map of crack surface of plain expansive soil sample; (**c**) 3D elevation map of improved expansive soil sample surface; (**d**) 3D elevation map of the crack surface of improved expansive soil sample.

Cracks of soil are caused by the decrease in bond strength between soil particles. Soil roughness can reflect the friction between soil particles, and the friction between particles can directly affect the bond strength. Therefore, to further explore the effect of soil roughness during the development of crack, in this study, the 3D elevations of the soil sample surface and the crack surface of plain expansive soil and the improved expansive soil after six cycles of drying and wetting were measured by the laser confocal 3D microscope, as shown in Figure 10.

From Figure 10, the surface elevation range of plain expansive soil and the improved expansive soil are between 43.31 and 207.58 μm and between 92.16 and 249.63 μm, respectively, while the elevation ranges of crack surface are between 61.28 and 364.29 μm and between 54.06 and 938.26 μm, respectively. The elevation of the crack surface is higher than the surface elevation of the soil sample, which indicates that the unevenness of the surface and the crack surface increases when the plain expansive soil is mixed with coal gangue powder.

The observed surface roughness of the soil sample is shown in Table 5.

It can be observed from Table 5 that the roughness of the crack surface of the improved expansive soil is larger than that of the plain expansive soil. Combined with the crack development, the crack rate of the improved expansive soil is smaller than that of the plain expansive soil. The reason is that the high valence cations in coal gangue powder react with the low valence cations in expansive soil to produce more stable cementation compounds. The internal meshing ability of the soil sample is improved, resulting in difficulty of crack expansion. The coal gangue particle itself is relatively rough, and the soil sample has an obvious tearing effect due to meshing in the cracking process. Therefore, the crack surface roughness of the improved expansive soil is greater than that of the plain expansive soil.

### 3.3. Micro-Pore Characteristic Analysis

In order to further study the mechanism of crack development in soil samples under different drying–wetting cycles from the perspective of micro-pores, mercury intrusion tests were carried out on soil samples under various drying–wetting cycles. The porosity, average pore diameter, total pore volume, cumulative pore volume, and cumulative pore density parameters of plain expansive soil and 9% gangue powder-improved expansive soil were obtained, and the pores with different sizes were divided, in order to study the change of pores in each range.

#### 3.3.1. Porosity and Average Pore Size

**Figure 11:**
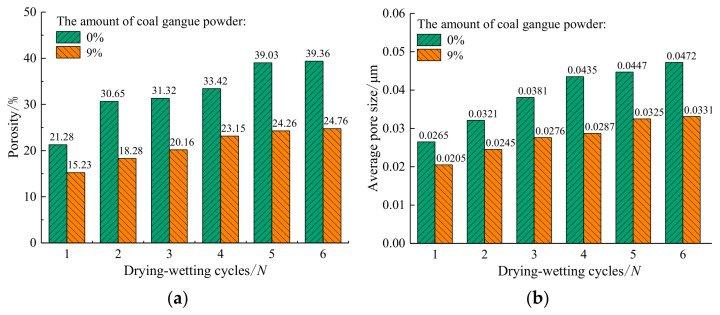
Changes of porosity and average pore diameter with the number of drying–wetting cycles: (**a**) variation of porosity with the number of drying –wetting cycles; (**b**) variation of average pore size with the number of drying–wetting cycles.

It can be observed from Figure 11a that the porosity of plain expansive soil increases with the increase in drying–wetting cycles. After two drying–wetting cycles, the porosity increased significantly compared with the first drying–wetting cycle. After six drying–wetting cycles, the porosity tended to be stable. The porosity of the improved expansive soil is small with the drying–wetting cycles. After six drying–wetting cycles, the porosity tends to be stable. Finally, the porosity of the improved expansive soil is 37% lower than that of the plain expansive soil. This shows that as the number of drying–wetting cycles increase, the number of pores inside the soil gradually increases as well, and the amount of the pores in the soil can be effectively reduced by coal gangue powder. It can be observed from Figure 11b that the average pore diameter of plain expansive soil and the improved expansive soil increases with the increase in drying–wetting cycles. Under each drying–wetting cycle, the average pore diameter of the improved expansive soil is significantly smaller than that of plain expansive soil. Finally, after six drying–wetting cycles, the average pore diameter of the improved expansive soil is 30% smaller than that of plain expansive soil. With the increase in porosity and average pore size, the internal structure of soil tends to be loose, and the micro-pores transform into macro-cracks. Coal gangue powder can reduce the porosity and average pore size of expansive soil, making the soil structure more stable.

#### 3.3.2. Pore Distribution Characteristics Analysis

**Figure 12:**
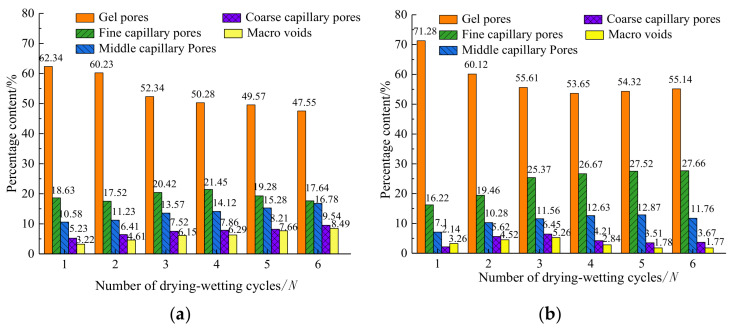
Variation of the percentage content of pores in plain expansive soil with the number of drying–wetting cycles: (**a**) plain expansive soil; (**b**) improved expansive soil.

In order to further study the distribution of pores under different drying and wetting cycles, it is necessary to divide the pore size. Nowadays, there are many methods to divide the pore size [38,39], but these methods are not suitable for expansive soil. As expansive soil belongs to fine-grained cohesive soil, and the internal micro-pore size is small, a more refined method should be used to divide the pore size. In this study, the pore size is divided into gel pores (d < 0.1 μm), fine capillary pores (0.11 μm < d < 2 μm), middle capillary pores (2 μm < d < 10 μm), coarse capillary pores (10 μm < d < 20 μm) and macro-voids (d > 20 μm), according to the method of pore size division by Yang et al. [40]. The pore types correspond to five types: pores in particles, pores between particles, pores in agglomerates and part of pores between particles, pores in agglomerates, pores between agglomerates, and part of pores in agglomerates. The histograms of the percentage of each pore of plain expansive soil and 9% coal gangue powder-improved expansive soil, changing with the number of drying–wetting cycles, is shown in Figure 12.

From Figure 12, we can see that when there are less than two cycles of drying–wetting, the proportion of gel pores (pores in particles) is the largest, and the gel pores of prime expansive soil gradually decrease, along with an increase in drying–wetting cycles. After four drying–wetting cycles, the fine capillary pores (pores between particles) reached the maximum. After five drying–wetting cycles, the fine capillary pores began to decrease, and the middle capillary pores (pores in agglomerates and part of pores between particles), coarse capillary pore (pores in agglomerates), and macro-voids (pores between agglomerates and part of pores in agglomerates) gradually increased as the drying–wetting cycles increased. This indicates that during the first four cycles of drying–wetting, the gel pores transformed to larger pores. After five cycles of drying–wetting, both the gel pores and the fine capillary pores transformed to larger pores. Compared with expansive soil, the gel pores of the improved expansive soil increase significantly under the drying–wetting cycles. With the increase in drying–wetting cycles, the gel pores decrease, while the fine capillary pores gradually increase, but the middle capillary pores, the coarse capillary pores, and the macro-voids are reduced. This further shows that the coal gangue powder can make the pore structure of the expansive soil more refined and reduce the larger types of pores. It is difficult to form group cracks, and macroscopically, it shows the decrease in cracks.

**Figure 13:**
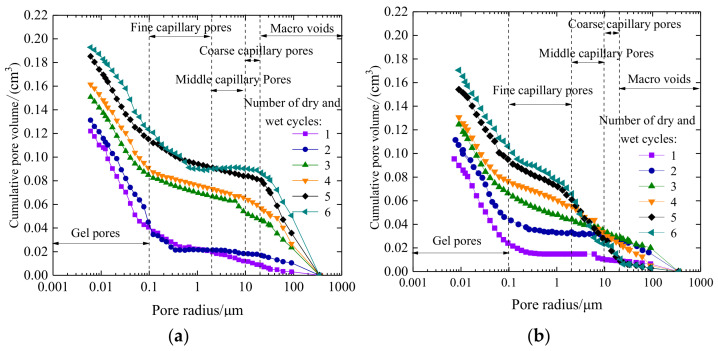
Cumulative pore volume distribution of soil samples under different drying–wetting cycles: (**a**) plain expansive soil; (**b**) improved expansive soil.

The cumulative pore volume distribution curves of plain expansive soil and 9% coal gangue powder-improved expansive soil under different drying–wetting cycles are shown in Figure 13.

It can be observed from Figure 13a that with the increase in drying–wetting cycles, the cumulative pore volume curve of plain expansive soil gradually moves upward. The cumulative pore volume curve of the first and second drying–wetting cycles is consistent, but after three drying–wetting cycles, the cumulative pore volume increases significantly, especially for a pore diameter greater than 0.1 μm. However, with the increase in the number of drying–wetting cycles, the volume of larger pores in the soil begins to increase, the effective stress in the soil decreases, and there is a decrease in the stability of the soil. It can be observed from Figure 13b that except for fine capillary pores, the cumulative pore volume curve of all types of pores decreases in the improved expansive soil. In the range of gel pores and fine capillary pores, the cumulative pore volume increases with the increase in wetting–drying cycles, and the curves become denser in the fine capillary pores. This indicates that coal gangue powder can reduce the influence of drying–wetting cycles on expansive soil. After two drying–wetting cycles, the cumulative pore volume of fine capillary pores, middle capillary pores, coarse capillary pores, and macro-voids is larger than that of plain expansive soil. This is because the coal gangue powder has not exchanged with the ions in the expansive soil in the early drying–wetting cycles yet, and the pore inside the coal gangue powder is caused by the pore itself. After three drying–wetting cycles, the cumulative pore volume in the range of fine capillary pores, middle capillary pores, and coarse capillary pores began to decrease significantly, and the curve became denser with the change of drying–wetting cycles. This is because after two drying–wetting cycles, the ion exchange reaction occurred between the high valence cations in coal gangue and the low valence cations in expansive soil. A more stable compound or gel was formed to fill the pores in the soil sample, thus reducing the porosity in the soil. The macro-cracks of soil samples in the drying and shrinkage process are caused by the continuous interconnection between large pores in the soil, and the coal gangue powder can reduce the cumulative volume of large pores from a micro-point of view, which reduces the connection range of large pores and macro-cracks.

**Figure 14:**
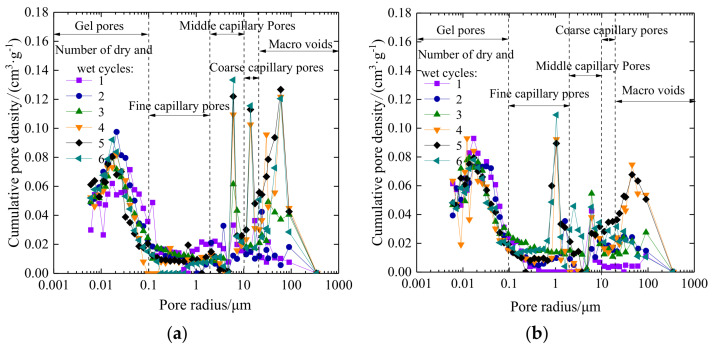
Cumulative pore density distribution of soil samples under different drying–wetting cycles: (**a**) plain expansive soil; (**b**) improved expansive soil.

The cumulative pore density distribution curves of plain expansive soil and the 9% coal gangue powder-improved expansive soil under different drying–wetting cycles are shown in Figure 14.

From Figure 14a, it can be observed that after three cycles of drying–wetting, the cumulative pore density curves begin to show wave peaks in the range of the middle capillary pores and macro-voids, which indicates the increase in pores in agglomerates and pores between agglomerates in the plain expansive soil. After four drying–wetting cycles, there are obvious wave peaks in the range of middle capillary pores, coarse capillary pores, and macro-voids. As the drying–wetting cycles increase, there is an increase in cumulative density of middle capillary pores and coarse capillary pores. However, the peak values of macro-voids in the fourth, fifth, and sixth drying–wetting cycles basically coincide. The curve changes of crack development parameters in the fourth, fifth, and sixth drying–wetting cycles are not significant; this is corresponding to the crack development curve, which further indicates that the macro-crack of the soil is produced by the connection and fusion of the large pores in the soil. It can be observed from Figure 14b that after the addition of 9% coal gangue powder into the expansive soil, the curves of gel pores change little, and there is a wave peak in the fine capillary pores. As the drying–wetting cycles increase, the peak value also increases gradually, indicating that the pores between particles increase, which is consistent with the cumulative pore volume curve analysis. In the range of middle capillary pores, coarse capillary pores, and macro-voids, the cumulative pore density curve is significantly lower than that of plain expansive soil. After four drying–wetting cycles, the double peak phenomenon disappears in the range of macro-voids, which indicates that coal gangue powder can make the internal pores of soil more refined, improve the stability, reduce the cumulative density of macro-voids, reduce the probability of connectivity, and reduce the macro-cracks.

### 3.4. Micro-Pore Mechanism of Expansive Soil Improved by Coal Gangue

**Figure 15:**
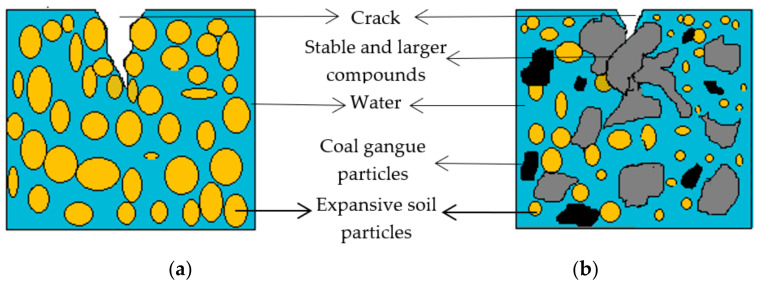
Schematic diagram of crack development of soil sample: (**a**) plain expansive soil; (**b**) improved expansive soil.

The above research demonstrates that after adding coal gangue powder into expansive soil, the parameters of soil cracks are significantly reduced compared with plain expansive soil, and the internal roughness is also increased. This is because the internal particles of expansive soil are smooth, and the internal resistance is relatively small in the cracking process, so the crack is easier to expand. After adding coal gangue powder, the high valence cations in coal gangue powder exchange with the low valence cations in expansive soil to form more stable and larger compounds. In the process of crack development, after the crack meets with the compound generated by coal gangue and expansive soil, the crack development path is blocked, which inhibits further development of the crack, which is one of the reasons why the crack parameters of the improved expansive soil are significantly lower than those of the plain expansive soil. The crack development diagram of plain expansive soil and the improved expansive soil is shown in Figure 15. The two-phase body consisting of solid and liquid soil is shown here, which is combined with the diagram of the pore to crack transformation in the back.

The generation of cracks in soil is caused by many factors. Next, we will explore and explain the mechanism of cracks caused by soil pores. Based on the test results obtained earlier in this study, the correlation between crack area ratio parameters and porosity parameters of plain expansive soil and the improved expansive soil under six drying–wetting cycles is analyzed, because these two parameters are more representative as a whole. The analysis results are shown in Table 6 and Table 7.

It can be observed from Table 6 and Table 7 that the correlation coefficients of porosity and crack area ratio of plain expansive soil and the improved expansive soil have reached more than 0.90, showing a strong correlation, and there are significant differences at their respective correlation levels, which is statistically significant. Therefore, the development of soil crack is closely related to the change of micro-pores.

**Figure 16:**
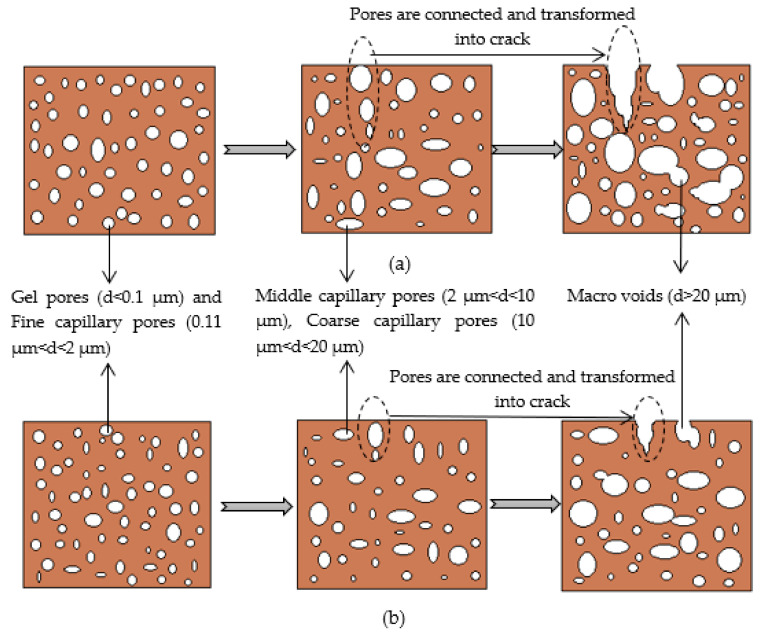
Schematic diagram of pores transformed into cracks under drying–wetting cycles: (**a**) plain expansive soil; (**b**) improved expansive soil.

Combined with the results of the previous mercury injection test, the evolution process from micro-pores to macro-cracks in the soil sample under the drying–wetting cycles is shown in Figure 16.

The analysis of the evolution mechanism from pore to crack of plain expansive soil is shown in Figure 16a. At the initial stage of drying–wetting cycles, the interior of plain expansive soil is mainly small pores, and there are no cracks at the initial stages. This is because the drying–wetting cycle has less impact on the soil and causes less damage to the internal structure. With the gradual increase in the number of drying–wetting cycles, due to the obvious water swelling and water loss shrinkage of plain expansive soil itself, the internal structure is damaged, the soil becomes loose, the internal pores also increase significantly, and the pores near the top of the soil begin to change into surface micro-cracks. With the further increase in the number of drying–wetting cycles, the internal structure of soil is further damaged, the internal macro-pores are further increased, the distance between pores is also reduced, and the connection and fusion phenomenon begins to appear between pores. The surface micro-crack formed by pores near the top of soil is also connected and fused with the internal macro-pores of soil, resulting in an obvious increase in cracks. The analysis of the evolution mechanism from the pore to the crack of the improved expansive soil is shown in Figure 16b. After the improvement of coal gangue, the cracks in expansive soil are significantly reduced. This is because coal gangue improves the stability of expansive soil, the ability to resist the effects of drying–wetting cycles is correspondingly improved, the internal structure of soil is not easy to be damaged, the soil is more dense, and there are relatively few internal macro-pores, which reduces the connection and fusion probability of internal macro-pores, so the cracks are also significantly reduced.

In conclusion, during the drying–wetting cycles, the plain expansive soil is damaged easily and the internal structure becomes loose. When cracks occur, the cracks will extend along the internal failure surface, resulting in the rapid increase in cracks. In addition, the connection and fusion of internal macro-pores will further increase the cracks. After the expansive soil is improved by coal gangue, the high valence cations in the coal gangue exchange with the low valence cations in the expansive soil to produce more stable compounds, which improves the stability of the soil, the internal structure is relatively stable, the internal macro-pores are relatively few, and the drying–wetting cycle resistance is improved. When the stable compounds are encountered at the crack tip, the crack will be blocked; in addition, the connection probability of internal macro-pores is reduced, so the crack is also significantly reduced.

## 4. Discussion

Expansive soil produces cracks easily in the drying–wetting cycle environment, because the expansive soil contains hydrophilic montmorillonite, illite, and other mineral components, which makes the expansive soil highly sensitive to water. Some scholars have improved the expansive soil with other materials (such as slag, fly ash, and lime) to reduce its cracks. The final results show the effectiveness of these methods [23,24,25]. Coal gangue contains more mineral components, such as low hydrophilic kaolinite and illite. After the expansive soil is improved with coal gangue, the proportion of montmorillonite will be relatively reduced; therefore, the hydrophilicity of expansive soil is reduced and its swelling and shrinkage properties are improved. Coal gangue can be used as road-building material and soil improvement material, which has been proved by other scholars [29]. The research on the development law and causes of cracks is helpful in solving engineering problems of expansive soil [6,7,8]. In this study, we found that the crack parameters of expansive soil increase with the increase in drying–wetting cycles. The crack parameters increase rapidly and then tend to be stable with an increase in time, which is consistent with the results of other scholars [41]. Coal gangue can obviously inhibit the crack development of expansive soil, the crack area rate decreases by 48.9%, and the crack germination time is also obviously delayed. Coal gangue can increase the roughness of expansive soil. The greater the internal roughness of soil, the less likely it is to produce cracks. This is because the greater the roughness, the greater the friction between particles, and the harder it is to separate. This is consistent with the results obtained by Fu et al. and Jocenei et al. [10]. During the development of cracks in expansive soil, the micro-pores in soil are also changing. This study shows that with the increase in the number of drying–wetting cycles, the average pore size and porosity in the soil gradually increase as well. This is consistent with the results obtained by Zhao et al. [42]. The average pore diameter and porosity of expansive soil were reduced by 37% and 30%, respectively, which may be caused by the interaction of mineral components in coal gangue and expansive soil. In this study, we divide the internal pores of soil in detail according to the size of diameter, which plays an important role in the study of macro-crack development mechanism. As the number of drying–wetting cycles increased, the gel pores and fine pores in the expansive soil changed to the middle capillary pores, large capillary pores, and macro-voids. The generation of the macro-crack is caused by the continuous connection and fusion of macro-pores in the soil. Coal gangue significantly reduces the proportion of macro-pores, cumulative pore volume, and cumulative pore density in expansive soil, so as to reduce the connection and fusion range and probability of macro-pores, and reduce the macro-cracks. After the correlation analysis of micro-pore parameters and macro-crack parameters, it is found that they have a high correlation. This is consistent with the research conclusion mentioned in the introduction [11,12].

The limitation of this study is that the improved expansive soil cannot be used in the subgrade construction of the high-grade highway. Another limitation is the long-term inhibitory effect of coal gangue on the crack development of expansive soil, which is because the road will be used for a long time after completion. We suggest that the inhibition effect of coal gangue on the crack development of expansive soil should be studied as long as possible. The interaction of mineral components in coal gangue and expansive soil may also affect crack development; this will be our research direction in the future.

## 5. Conclusions

A series of laboratory tests on the coal gangue-improved expansive soil under drying–wetting cycles; conditions are carried out, and the macro-crack development law and micro-pore evolution mechanisms are further explored. The conclusions are as follows:

The crack area ratio, total crack length, and average crack width of plain expansive soil increases with the increase in drying–wetting cycles. After adding 9% coal gangue powder into plain expansive soil, the crack parameters and crack development speed significantly decrease, and the crack germination time is delayed by at least 60 min.

The roughness of soil has a great influence on the development of soil cracks. The larger the roughness, the less prone to cracks. Coal gangue powder can effectively increase the roughness of expansive soil and reduce the cracks.

The results show that the porosity and average pore diameter of plain expansive soil increase under the action of drying–wetting cycles. After six drying–wetting cycles, the porosity and average pore diameter of the improved expansive soil decreases by 37% and 30%, respectively, compared with the plain expansive soil.

Through the comparative analysis of the cumulative pore volume and cumulative pore density parameters of soil samples under different drying–wetting cycles, it is found that the cracks are caused by the continuous connection and fusion of macro-voids in the soil. Adding 9% coal gangue powder into expansive soil can significantly reduce the proportion of macro-voids, cumulative pore volume, and cumulative pore density. Therefore, the connection and fusion range and connection probability of macro-pores are also significantly reduced, and the macro-cracks are also reduced.

## Figures and Tables

**Table 1:** Basic indexes of expansive soil.

Liquid Limit/%	Plastic Limit/%	Plasticity Index	Loss on Ignition Rate/%	Dry Density/(g·cm^−3^)	Free Expansion Rate/%
52.3	27.7	24.6	13.5	1.61	49

**Table 2:** Basic Chemical constituents of expansive soil/%.

SiO_2_	Al_2_O_3_	Fe_2_O_3_	MgO	CaO	Na_2_O	K_2_O	P_2_O_5_
64.3	13.34	2.63	2.76	0.88	1.73	0.88	0.06

**Table 3:** Basic indexes of coal gangue.

Free Expansion Rate/%	Water Absorption/%	Crushing Value/%	Non-Uniformity Coefficient	Curvature Coefficient	Loss on Ignition Rate/%
13.7	0.7	20.5	3.4	2.2	14.2

**Table 4:** Basic chemical constituents of coal gangue/%.

SiO_2_	Al_2_O_3_	Fe_2_O_3_	CaO	MgO	C
62.1	24.8	4.3	1.9	1.3	3

**Table 5:** Observation surface roughness of soil sample.

Proportion of Coal Gangue Powder/%	Surface Roughness of Soil Sample/μm	Roughness of Soil Sample Crack Surface/μm
0	13.69	48.91
9	14.36	95.45

**Table 6:** Correlation between porosity and crack area ratio of plain expansive soil.

Parameter	Pearson Correlation	Sig. (2-Tailed)	N
Porosity	1	-	6
Crack area ratio	0.910 *	0.012	6

* At the level of 0.05 (2-tailed), the correlation was significant.

**Table 7:** Correlation between porosity and crack area ratio of improved expansive soil.

Parameter	Pearson Correlation	Sig. (2-Tailed)	N
Porosity	1	-	6
Crack area ratio	0.977 **	0.001	6

** At the level of 0.01 (2-tailed), the correlation was significant.

## Data Availability

The data used to support the findings of this study are included within the article.
